# Effect of the number of coronavirus disease 2019 (COVID-19) vaccination shots on the occurrence of pneumonia, severe pneumonia, and death in SARS-CoV-2-infected patients

**DOI:** 10.3389/fpubh.2023.1330106

**Published:** 2024-01-08

**Authors:** Shijun Xin, Wei Chen, Qilin Yu, Li Gao, Genjie Lu

**Affiliations:** Department of Blood Transfusion, Ningbo Medical Center Lihuili Hospital, Ningbo University, Ningbo, China

**Keywords:** COVID-19, vaccination, pneumonia, severe pneumonia, death

## Abstract

**Background:**

Coronavirus disease (COVID-19) has posed a significant threat to the lives and health of people worldwide since its onset in 2019. However, the relationship between the number of vaccination shots and the severity of SARS-CoV-2 infection in Chinese patients remains unclear.

**Methods:**

We retrospectively collected information from 829 patients infected with SARS-CoV-2 in Ningbo Medical Center Lihuili Hospital from December 05, 2022 to March 31, 2023, then divided them into four groups based on the severity of pneumonia. Last, we compared the difference in the number of shots of COVID-19 vaccine between the four groups, considering potential confounding factors using univariate and multivariate logistic regression.

**Results:**

Vaccination with two and three doses was positively associated with low prevalence of pneumonia and severe pneumonia both in crude and optimal models, while only three doses of the vaccine was correlated with low prevalence of death in SARS-CoV-2-infected patients. In optimal models, male SARS-CoV-2-infected individuals with advanced age were positively associated with high prevalence of pneumonia, severe pneumonia, and death; comorbidity with hypertension (OR = 2.532, *p* < 0.001) was positively associated with high prevalence of pneumonia (OR = 2.532, *p* < 0.001); and comorbidity with diabetes was positively associated with high prevalence of death (OR = 1.856, *p* = 0.011). However, this is a cross-sectional study and the causal relationships need to be further studied.

**Conclusion:**

One dose of vaccine may not have a protective effect against pneumonia, severe pneumonia, and death; more than one dose of vaccine is an independent protective factor for pneumonia and severe pneumonia; and three doses of vaccine is an independent protective factor for death.

## Introduction

Coronavirus disease (COVID-19) was caused by the severe acute respiratory syndrome coronavirus 2 (SARS-CoV-2). Since COVID-19 swept the globe in 2019, it has presented a huge challenge to human life and health globally, greatly thwarting human economic and social development, and healthcare systems around the world are under unprecedented pressure (1). In particular, with the mutation of the SARS-CoV-2 virus, the Omicron variant became globally prevalent for a time (2), putting people at higher risk of reinfection (3), which presented yet another challenge.

Coronaviruses (CoVs) are a highly diverse family of envelope-positive single-stranded RNA viruses characterized by high rates of genetic recombination and mutation leading to their ecological diversity (4). On the basis of genome sequence and serologic response, they can be classified into alphacoronavirus, betacoronavirus, gammacoronavirus, and deltacoronavirus. SARS-CoV-2 belongs to betacoronavirus. SARS-CoV-2 infection involves multiple cell surface receptors and multiple pathways (5–7), and it is mainly transmitted through the respiratory tract, and the clinical manifestations of SARS-CoV-2 infections vary widely in clinical manifestations, ranging from asymptomatic or mild infections to pneumonia, and can also develop into life-threatening respiratory diseases in severe cases (8, 9). Currently, the treatment of COVID-19 has a corresponding approach according to different clinical syndromes (10). At the same time, many anti-COVID-19 drugs are being developed (11). Nevertheless, Long COVID occurs in at least 10% of SARS-CoV-2 infections and is an often debilitating illness (12). Therefore, the prevention of COVID-19 is still the most effective means of safeguarding the lives and health of the public against this disease.

Scientists around the world have been working on vaccine research and development for as long as SARS-CoV-2 has been recognized in humans (13). The process of developing and producing a vaccine for COVID-19 was further accelerated with the introduction of the COVID-19 Vaccines Global Access (COVAX). Thanks to this program, as of August 2023, 70.48% of the world population has received at least one dose of a COVID-19 vaccine, and China reached its first peak for the SARS-CoV-2 vaccine in June 2020 (14). Nonetheless, there is still a portion of the population that is not up-to-date or not fully vaccinated for various reasons, such as uneven distribution of vaccine resources, vaccine reactions, and a lack of knowledge about vaccines (15–17). However, strict adherence to the vaccination regimen is essential for the vaccine to produce optimal protection (18). Booster immunization involves the administration of an additional dose of vaccine after the initial vaccination to induce immune memory and improve protection against the corresponding viral infection. Several studies have also emphasized the importance of booster shots (19–22). Nowadays, although the pandemic trend of COVID-19 has passed, the number of new infections still fluctuates within a certain range globally (14), so we still cannot completely let down our guard.

The importance of the number of COVID-19 vaccinations, especially complete vaccination courses, has been reported in a few studies (8, 23–25). There have also been several studies conducted in the Chinese population. A study based in Yunan, China, reported that the vaccine reduced the risk of pneumonia and severe pneumonia after SARS-CoV-2 infection (26). A study based in the Hong Kong Special Administrative Region, China, reported that the COVID-19 vaccine reduced the risk of death (27). However, there is a lack of broader population-based studies on the number of shots of COVID-19 vaccine and the risk of pneumonia, severe pneumonia, and death in patients with new SARS-CoV-2 virus infections.

## Method

### Study design and population

In this study, we retrospectively collected data from December 05, 2022 to March 31, 2023. A total of 837 COVID-19 cases were admitted to Ningbo Medical Center Lihuili Hospital during the period, totaling 829 cases after the exclusion of 8 cases of underage patients, including 456 male and 373 female patients. Patients attending COVID-19 were categorized into non-pneumonia versus pneumonia and non-severe pneumonia versus severe pneumonia groups according to clinical diagnosis. Patients were also categorized into non-death and death groups. This study was approved by the ethics committee of Ningbo Medical Center Lihuili Hospital (Approval No: Li Huili Hospital Ethics Review 2023 Research No. 082).

### Data collection and definitions of covariates

The diagnosis of COVID-19 and basic patient information, including gender, age, BMI, diabetes mellitus, hypertension, and malignant tumors, were collected from electronic medical records (EMR) (KINGT Software, Ningbo Jintang Software Co., Ltd., China). Vaccination information, including the number of vaccination shots and types, was collected from the SaaS Yunjinmiao vaccination system of Zhejiang Province (Shensu Science & Technology (Suzhou) Co., Ltd., China). Age and BMI were categorized according to the median, which was 71 for age and 23.07 for BMI.

### Measurement of outcomes

The diagnosis of COVID-19 and the definition of the severity of pneumonia were based on examination by doctors according to the Diagnosis and Treatment Protocol for COVID-19 (Trial Version 9) (28).

### Statistical analysis

Statistical analyses were performed with SPSS 22.0 and R.4.2.0. Continuous variables are described as mean and standard deviation and categorical variables are described as frequency and percentage. First, univariate logistic regression was performed to calculate the odds ratio (OR) of the number of vaccination shots, and the differences between the groups categorized by pneumonia, severe pneumonia, and death after SARS-CoV-2 infection were compared. Participants without pneumonia were treated as the reference group. The indicators with statistical significance (*p* < 0.05) in univariate logistic regression were adopted as the potential confounding factors in the multivariate logistic regression model to further examine the association between the number of vaccination shots and the severity of pneumonia after SARS-CoV-2 Infection. Last, we removed the variables with *p* > 0.05 in multivariate logistic models and re-conducted the multivariate logistic regression to fit the optimal model. All reported probabilities (*p*-values) were two-sided, with *p* < 0.05 considered statistically significant.

## Result

### Basic characters of participants

The general characteristics of the study population are summarized in Table 1. We finally included 829 participants in the analysis, of which 456(55.00%) were male and 373(45.00%) were female. Of these, 404 participants were aged greater than or equal to 71 years, representing 51.27% of the study population. BMI greater than or equal to 23.07 was 410(49.46%), 411(49.58%) were hypertensive, 212(25.57%) were diabetic, and 146(17.61%) had malignancy. The number of people vaccinated with 0 to 3 COVID-19 vaccine doses were 303 (36.55%), 83 (10.01%), 147 (17.73%), and 296 (35.71%), respectively. As for the type of vaccine, the number of people vaccinated with inactivated vaccine for all injections was 440, which is 53.08% of the total number of people in the study and 83.65% of the number of people vaccinated. The number of people vaccinated with adenovirus vector vaccine or recombinant protein vaccine for all injections and the number of people vaccinated with a mixture of vaccines of different attributes were 31 and 55, respectively, accounting for 3.74% and 6.63% of the total number of people in the study, respectively.

**Table 1:** Characteristics of participants infected with COVID-19 in the study.

	*N*(%)	Pneumonia		Severe pneumonia		Mortality	
		No	Yes	No	Yes	No	Yes
All participants	829(100.00%)	135	694	621	208	744	85
Sex							
Male	456(55.00%)	51	405	315	141	393	63
Female	373(45.00%)	84	289	306	67	351	22
Age							
<71	404(48.73%)	103	301	341	63	373	31
≥71	425(51.27%)	32	393	280	145	371	54
Body mass index (BMI)							
<23.07	410(49.46%)	77	333	307	103	370	40
≥23.07	419(50.54%)	58	361	314	105	374	45
Hypertension							
No	418(50.42%)	101	317	332	86	376	42
Yes	411(49.58%)	34	377	289	122	368	43
Diabetes							
No	617(74.43%)	115	502	478	139	567	50
Yes	212(25.57%)	20	192	143	69	177	35
Cancer							
No	683(82.39%)	113	570	506	177	616	67
Yes	146(17.61%)	22	192	115	31	128	18
Number of vaccinations							
0	303(36.55%)	33	270	201	102	259	44
1	83(10.01%)	7	76	57	26	74	9
2	147(17.73%)	35	112	116	31	132	15
3	296(35.71%)	60	236	247	49	229	17
Vaccine type							
Unvaccinated	303(36.55%)	33	270	201	102	259	44
Inactivated vaccine	440(53.08%)	86	354	356	84	406	34
Others	31(3.74%)	5	26	20	11	29	2
Mixed inoculation	55(6.63%)	11	44	44	11	50	5

Others include recombinant protein vaccine and adenovirus vector vaccine.

### Relationship between number of COVID-19 vaccination shots and pneumonia

**Figure 1:**
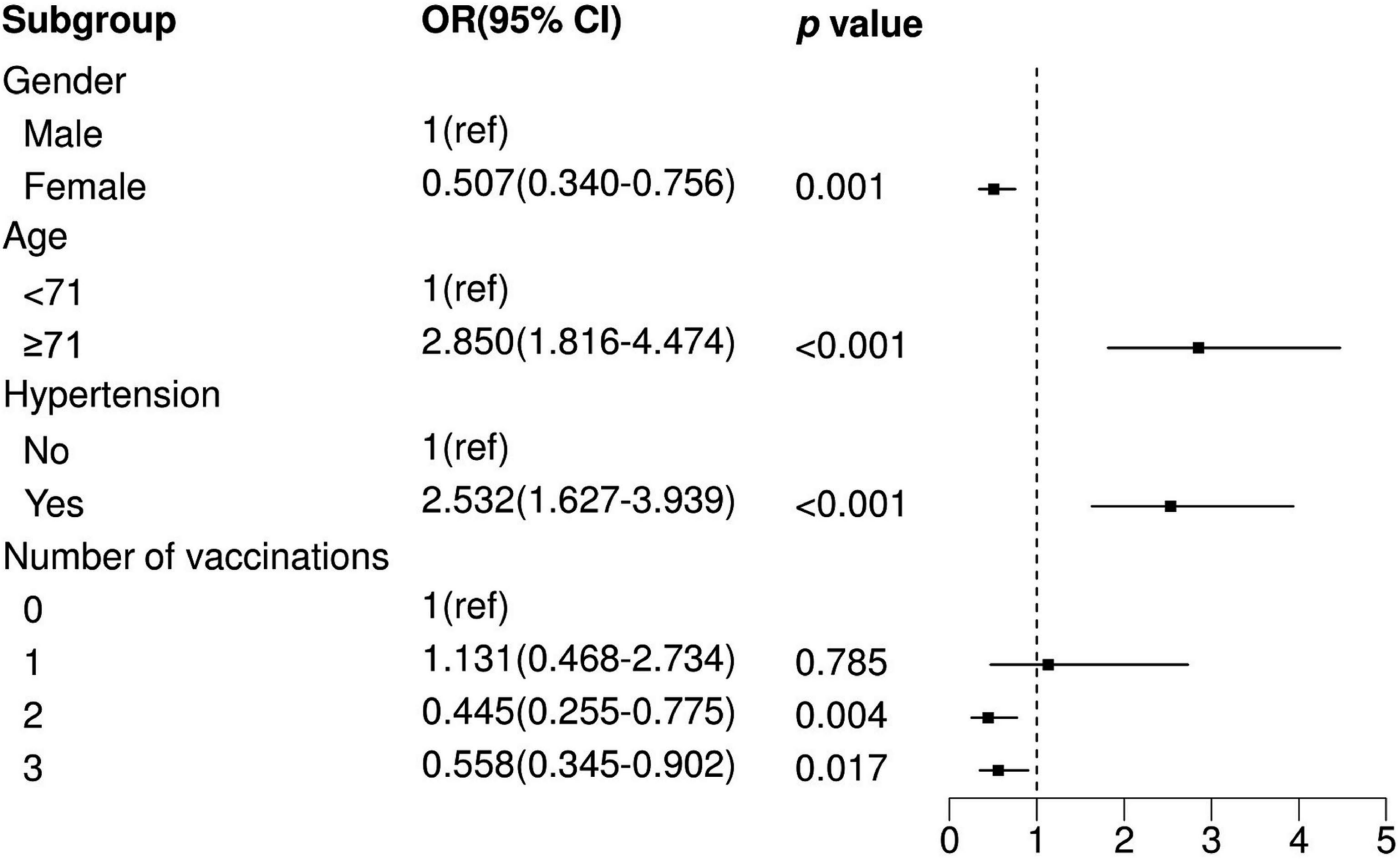
Multivariate logistic regression analysis between non-pneumonia and pneumonia. OR, Odds Ratio; CI, Confidence Interval; ref., Reference.

We observed that two and three vaccination shots were positively correlated with low prevalence of pneumonia in SARS-CoV-2-infected patients in a univariable logistic model (Two shots: OR = 0.391, *p* < 0.001; Three shots: OR = 0.481, *p* = 0.002), while one shot of vaccination was not significantly associated with pneumonia (Table 2). In addition, other factors, namely, gender, age, and comorbidity, showed significant differences between the non-pneumonia and pneumonia groups (Table 2). After performing multivariable logistic regression and retaining nominally significant variables, two and three vaccination shots remained significant, and their OR were 0.445 and 0.558 in the optimal model (Two shots: *p* = 0.004; Three shots: *p* = 0.017) (Figure 1). Moreover, male patients (OR = 1.343, *p* < 0.001), patients with age ≥ 71 (OR = 2.850, *p* < 0.001) or those that were comorbid with hypertension (OR = 2.532, *p* < 0.001) were positively correlated with high prevalence of pneumonia.

**Table 2:** Crude odds ratio results between non-pneumonia and pneumonia.

	Non-pneumonia	Pneumonia	OR (95% CI)	*p*-value
All participants	135	694	–	–
Sex	51	405	1(ref)	
Male	84	289	0.433(0.297–0.633)	<0.001
Female				
Age				
<71	103	301	1(ref)	
≥71	32	393	4.203(2.750–6.423)	<0.001
Body mass index (BMI)				
<23.07	77	333	1(ref)	
≥23.07	58	361	1.439(0.992–2.088)	0.055
Hypertension				
No	101	317	1(ref)	
Yes	34	377	3.533(2.330–5.357)	<0.001
Diabetes				
No	115	502	1(ref)	
Yes	20	192	2.199(1.330–3.637)	0.002
Cancer				
No	113	570	1(ref)	
Yes	22	192	1.117(0.680–1.835)	0.661
Number of vaccinations				
0	33	270	1(ref)	
1	7	76	1.327(0.565–3.118)	0.516
2	35	112	0.391(0.232–0.661)	<0.001
3	60	236	0.481(0.304–0.761)	0.002
Vaccine type				
Unvaccinated	33	270	1(ref)	
Inactivated vaccine	86	354	0.503(0.327–0.775)	0.002
Others	5	26	0.636(0.228–1.768)	0.385
Mixed inoculation	11	44	0.489(0.230–1.038)	0.063

OR, Odds Ratio; CI, Confidence Interval; ref, Reference. Others include recombinant protein vaccine and adenovirus vector vaccine.

### Relationship between number of vaccination shots and severe pneumonia

**Figure 2:**
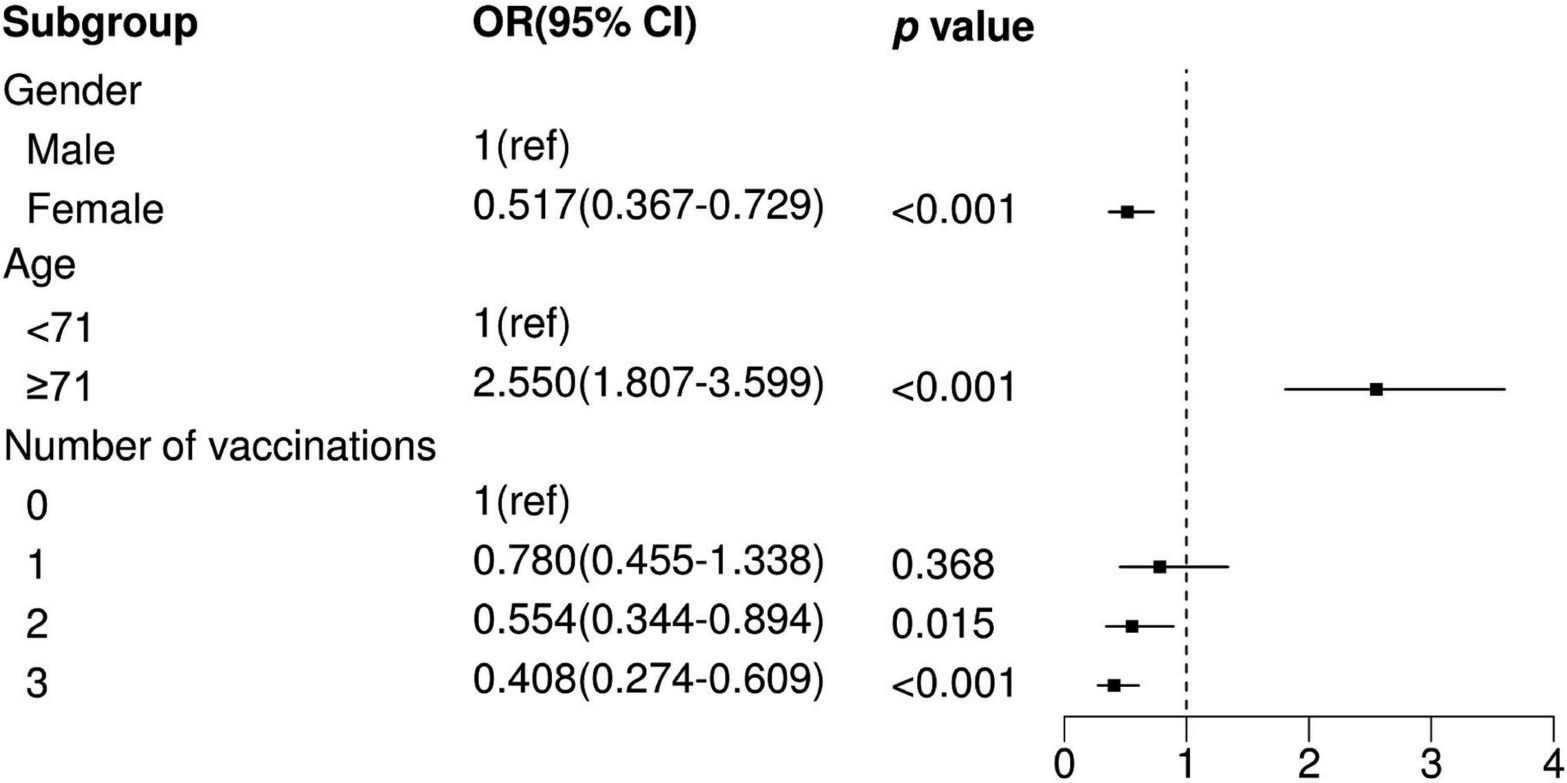
Multivariate logistic regression analysis between non-severe pneumonia and pneumonia. OR, Odds Ratio; CI, Confidence Interval; ref., Reference.

We observed that two and three vaccination shots were positively correlated with low prevalence of severe pneumonia in SARS-CoV-2-infected patients (Two shots: OR = 0.527, *p* = 0.007; Three shots: OR = 0.391, *p* < 0.001) in univariable logistic analysis (Table 3). In addition, other factors, namely, gender, age, hypertension, and diabetes, showed significant differences between the severe pneumonia and pneumonia groups (Table 3). After performing multivariable logistic regression and retaining nominally significant variables, two and three vaccination shots were still significant in the optimal model (Figure 2; Two shots: OR = 0.554, *p* = 0.015; Three shots: OR = 0.408, *p* < 0.001). Moreover, male patients (OR = 1.332, *p* < 0.001), patients with age ≥ 71 (OR = 2.550, *p* < 0.001) were also positively correlated with high prevalence of severe pneumonia.

**Table 3:** Crude odds ratio results between non-severe pneumonia and severe pneumonia.

	Non-severe pneumonia	Severe pneumonia	OR (95% CI)	*p*-value
All participants	621	208	–	–
Sex				
Male	315	141	1(ref)	
Female	306	67	0.489(0.351–0.681)	<0.001
Age				
<71	341	63	1(ref)	
≥71	280	145	2.803(2.004–3.920)	<0.001
Body mass index (BMI)				
<23.07	307	103	1(ref)	
≥23.07	314	105	0.997(0.728–1.364)	0.983
Hypertension				
No	332	86	1(ref)	
Yes	289	122	1.630(1.186–2.239)	0.003
Diabetes				
No	478	139	1(ref)	
Yes	143	69	1.659(1.177–2.340)	0.004
Cancer				
No	506	177	1(ref)	
Yes	115	31	0.771(0.500–1.187)	0.237
Number of vaccinations				
0	201	102	1(ref)	
1	57	26	0.899(0.534–1.514)	0.689
2	116	31	0.527(0.332–0.836)	0.007
3	247	49	0.391(0.265–0.576)	<0.001
Vaccine type				
Unvaccinated	201	102	1(ref)	
Inactivated vaccine	356	84	0.465(0.332–0.651)	<0.001
Others	20	11	1.084(0.500–2.349)	0.838
Mixed inoculation	44	11	0.493(0.244–0.994)	0.048

OR, Odds Ratio; CI, Confidence Interval; ref, Reference. Others include recombinant protein vaccine and adenovirus vector vaccine.

### Relationship between death and number of vaccination shots

**Figure 3:**
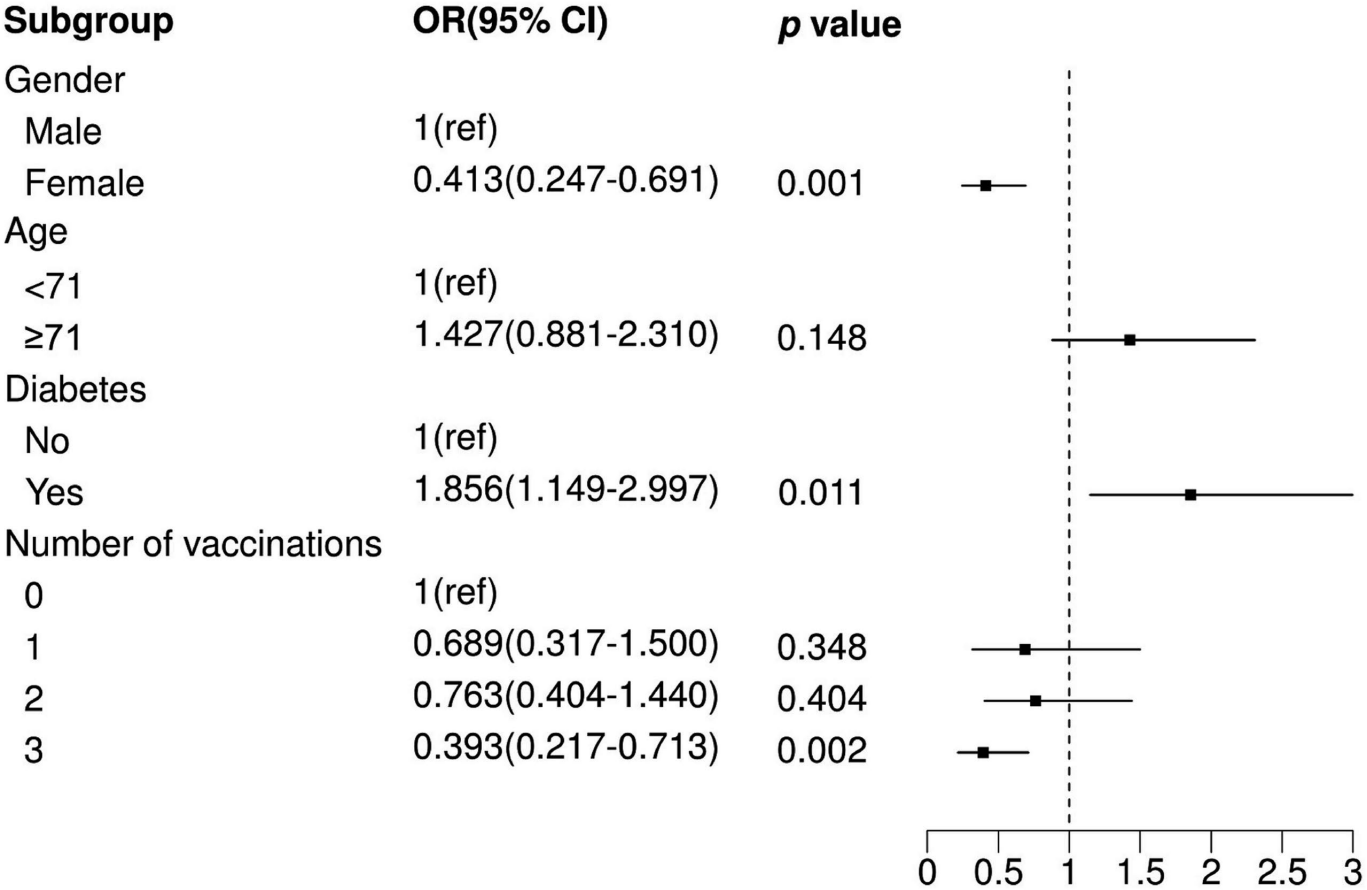
Multivariate logistic regression analysis between non-mortality and mortality. OR, Odds Ratio; CI, Confidence Interval; ref., Reference.

To further explore the relationship between the number of vaccination shots and death in SARS-CoV-2-infected patients, we adopted the same analytical approach as previously mentioned. The results showed that only three vaccination shots were positively correlated with low prevalence of death in SARS-CoV-2-infected patients (Three shots: OR = 0.359, *p* = 0.001) in a univariable logistic model (Table 4) and were still significant in the optimal model (Figure 3; Three shots: OR = 0.393, *p* = 0.002). Moreover, individuals who were comorbid with diabetes (OR = 1.856, *p* = 0.011) and male (OR = 1.468, *p* = 0.001) were also positively correlated with high prevalence of death in SARS-CoV-2-infected patients.

**Table 4:** Crude odds ratio results between non-mortality and severe mortality.

	Non-mortality	Mortality	OR (95% CI)	*p*-value
All participants	744	85	–	–
Sex				
Male	393	63	1(ref)	
Female	351	22	0.391(0.236–0.649)	<0.001
Age				
<71	373	31	1(ref)	
≥71	371	54	1.751(1.101–2.787)	0.018
Body mass index (BMI)				
<23.07	370	40	1(ref)	
≥23.07	374	45	1.113(0.710–1.745)	0.641
Hypertension				
No	376	42	1(ref)	
Yes	368	43	1.046(0.668–1.639)	0.844
Diabetes				
No	567	50	1(ref)	
Yes	177	35	2.242(1.410–3.565)	0.001
Cancer				
No	616	67	1(ref)	
Yes	128	18	1.293(0.743–2.250)	0.363
Number of vaccinations				
0	259	44	1(ref)	
1	74	9	0.716(0.334–1.534)	0.390
2	132	15	0.669(0.359–1.246)	0.205
3	229	17	0.359(0.200–0.644)	0.001
Vaccine type				
Unvaccinated	259	44	1(ref)	
Inactivated vaccine	406	34	0.493(0.307–0.792)	0.003
Others	29	2	0.406(0.094–1.762)	0.229
Mixed inoculation	50	5	0.589(0.222–1.558)	0.286

OR, Odds Ratio; CI, Confidence Interval; ref, Reference. Others include recombinant protein vaccine and adenovirus vector vaccine.

## Discussion

In this study, we retrospectively collected information about SARS-CoV-2-infected patients to investigate the relationships between the number of vaccination shots and the severity of pneumonia. Our main finding suggested that two or three vaccination shots may decrease the risk of pneumonia and severe pneumonia in SARS-CoV-2-infected patients, while only three vaccination shots may reduce the risk of death after COVID-19 infection, which highlights the importance of multiple vaccinations. Our findings also illustrate that one dose of COVID-19 vaccine is not significantly protective in SARS-CoV-2-infected patients, whereas more than one dose of vaccine may be effective in reducing the incidence of pneumonia and severe pneumonia in SARS-CoV-2-infected patients. However, three doses are necessary for the vaccine to be protective against death. This may be closely linked to the production of sufficient amounts of protective antibodies (29).

There are three main types of COVID-19 vaccines in China. The first is inactivated vaccines, including three inactivated vaccines produced by Sinopharm Zhongsheng (Beijing), Sinopharm Zhongsheng (Wuhan), and Beijing Kexing Zhongwei (Beijing); these are the vaccines administered to the majority of the population in China. The second is adenovirus vector vaccine type 5, produced by Tianjin Kangxinuo Company. Third is the recombinant protein vaccine (mRNA), a recombinant new coronavirus vaccine (CHO cells) produced by Anhui Zhifei. Three injections are required for the inactivated vaccine and the recombinant protein vaccine, and the adenovirus vector vaccine requires one injection. The different types of vaccines mentioned here did not differ in effect in our study. It can be seen that the production process of the vaccine does not make a difference in the level of protection of SARS-CoV-2-infected patients.

Our study found that men are more likely than women to have adverse outcomes after SARS-CoV-2 infection, which is also consistent with previous studies (30–33). These differences may be strongly related to factors such as gender-specific behaviors and biological pathways related to sex hormones and SARS-CoV-2 infection (31, 34). Several studies have also shown that women have stronger immune responses to vaccines than men, which may be one of the reasons why women have stronger immune efficacy when infected with SARS-CoV-2 (35, 36). In addition, there are different degrees of age-related declines in immune responses in older adult patients due to the onset of immune senescence (37, 38), which greatly elevates the risk of patients developing severe COVID-19 (39), which also corroborates the conclusions we obtained.

The presence of comorbidities is considered to be an important risk factor for exacerbating adverse outcomes in SARS-CoV-2-infected patients (40), and in a study by Guangtong Deng et al., hypertension, diabetes mellitus, and cancer were all reported to be risk factors for mortality in COVID-19 patients (41). It has been reported that genetically predicted high IGF-1 levels are associated with reduced COVID-19 susceptibility and risk of hospitalization (42), and diabetic patients tend to have reduced blood levels of IGF-1, which may be an important reason why diabetes is a risk factor for death in COVID-19 patients. Interestingly, however, our study only identified hypertension and diabetes mellitus as risk factors for pneumonia and death from infection, respectively, and there was no effect of cancer on the occurrence of pneumonia, severe pneumonia, and death after SARS-CoV-2 infection. This finding is not entirely consistent with the above report, and we speculate that the possible reason for this is that, in addition to the small number of cases included in the analysis compared to other studies, 83 of the 146 SARS-CoV-2-infected cases with cancer were also vaccinated with COVID-19 vaccine, and the vaccine still has some protective effect in these patients. This conjecture is supported by the fact that the majority of cancer patients found in the Luigi Cavanna et al. meta-analysis had an immunogenic response to COVID-19 vaccination (43).

Nowadays, although we have safely passed the most menacing stage of COVID-19, the protective effect of the SARS-CoV-2 vaccine is weakening over time (44), and there are still many individuals experiencing multiple infections due to SARS-CoV-2. In our study, as well as in previous studies, older adults with the presence of underlying diseases remain the most prominent victims of COVID-19 (40, 45, 46). Therefore, now, half a year after the peak of COVID-19, we still call on the public to strengthen awareness of prevention and to receive timely vaccination or catch-up vaccination when the situation permits in order to minimize the damage to health caused by COVID-19.

However, our study did have some limitations. First, as this is a cross-sectional study, the causal effect of the number of vaccination shots on the severity of pneumonia in SARS-CoV-2-infected patients could not be inferred. Second, although some potential confounding factors were considered in our study, other unmeasured confounders could affect the relationship between vaccination shots and the outcomes. Last, our study is not a multicenter study, which may limit the representativeness of the population.

## Conclusion

Our findings show that one dose of vaccine may not have a protective effect against pneumonia, while two and three vaccination shots were an independent protective factor for pneumonia and severe pneumonia, and three doses of vaccine was an independent protective factor for death.

## Data availability statement

The original contributions presented in the study are included in the article, further inquiries can be directed to the corresponding author.

## Ethics statement

The studies involving humans were approved by the ethics committee of Ningbo Medical Center Lihuili Hospital. The studies were conducted in accordance with the local legislation and institutional requirements. Written informed consent for participation was not required from the participants or the participants' legal guardians/next of kin in accordance with the national legislation and institutional requirements.

## Author contributions

SX: Data curation, Formal analysis, Investigation, Methodology, Software, Visualization, Writing – original draft. WC: Investigation, Writing – review & editing. QY: Investigation, Writing – review & editing. LG: Investigation, Writing – review & editing. GL: Data curation, Investigation, Supervision, Writing – review & editing.
